# Identification of a Variant in *APOB* Gene as a Major Cause of Hypobetalipoproteinemia in Lebanese Families

**DOI:** 10.3390/metabo11090564

**Published:** 2021-08-24

**Authors:** Carine Ayoub, Yara Azar, Yara Abou-Khalil, Youmna Ghaleb, Sandy Elbitar, Georges Halaby, Selim Jambart, Marie-Hélène Gannagé-Yared, Cesar Yaghi, Carole Saade Riachy, Ralph El Khoury, Jean-Pierre Rabès, Mathilde Varret, Catherine Boileau, Petra El Khoury, Marianne Abifadel

**Affiliations:** 1 Laboratory of Biochemistry and Molecular Therapeutics (LBTM), Faculty of Pharmacy, Pôle Technologie-Santé, Saint Joseph University of Beirut, Beirut 17-5208, Lebanon; carine.ayoub@net.usj.edu.lb (C.A.); yara.azar@inserm.fr (Y.A.); yara.abou-khalil@inserm.fr (Y.A.-K.); youmna.ghaleb@inserm.fr (Y.G.); sandy.el-bitar@inserm.fr (S.E.); 2 Laboratory for Vascular Translational Science (LVTS), INSERM U1148, Bichat Hospital, F-75018 Paris, France; jean-pierre.rabes@aphp.fr (J.-P.R.); mathilde.varret@inserm.fr (M.V.); catherine.boileau@inserm.fr (C.B.); 3 Centre Hospitalo-Universitaire Xavier Bichat, Université de Paris, F-75018 Paris, France; 4 Faculty of Medicine, Saint Joseph University of Beirut, Beirut 17-5208, Lebanon; mjhalaby@wise.net.lb (G.H.); selim.jambart@gmail.com (S.J.); mariehelene.yared@usj.edu.lb (M.-H.G.-Y.); cesaryaghi@gmail.com (C.Y.); carolesaaderiachy@gmail.com (C.S.R.); ralphelkhoury@gmail.com (R.E.K.); 5 Hotel Dieu de France of Beirut University Hospital, Beirut 166830, Lebanon; 6 Biochemistry and Molecular Genetics Laboratory, AP-HP, Université Paris-Saclay, Ambroise Paré Hospital, Boulogne Billancourt, UVSQ, UFR Simone Veil—Santé, F-78180 Montigny-Le-Bretonneux, France; 7 Genetics Department, AP-HP, Bichat Hospital, F-75018 Paris, France

**Keywords:** familial hypobetalipoproteinemia, low-density lipoprotein cholesterol, *APOB* gene, PCSK9, non-alcoholic fatty liver, diabetes, Lebanon, Middle East

## Abstract

Familial hypobetalipoproteinemia (FHBL) is a codominant genetic disorder characterized by reduced plasma levels of low-density lipoprotein cholesterol and apolipoprotein B. To our knowledge, no study on FHBL in Lebanon and the Middle East region has been reported. Therefore, we conducted genetic studies in unrelated families and probands of Lebanese origin presenting with FHBL, in order to identify the causes of this disease. We found that 71% of the recruited probands and their affected relatives were heterozygous for the p.(Arg490Trp) variant in the *APOB* gene. Haplotype analysis showed that these patients presented the same mutant haplotype. Moreover, there was a decrease in plasma levels of PCSK9 in affected individuals compared to the non-affected and a significant positive correlation between circulating PCSK9 and ApoB levels in all studied probands and their family members. Some of the p.(Arg490Trp) carriers suffered from diabetes, hepatic steatosis or neurological problems. In conclusion, the p.(Arg490Trp) pathogenic variant seems a cause of FHBL in patients from Lebanese origin, accounting for approximately 70% of the probands with FHBL presumably as a result of a founder mutation in Lebanon. This study is crucial to guide the early diagnosis, management and prevention of the associated complications of this disease.

## 1. Introduction

Familial hypobetalipoproteinemia (FHBL1, OMIM #615558) is the most common monogenic form of primary hypobetalipoproteinemia [1,2]. It is a codominant genetic disorder affecting lipoprotein metabolism, characterized by reduced plasma levels of total cholesterol (TC), low density lipoprotein-cholesterol (LDL-C) and apolipoprotein B (ApoB) below the fifth percentile of the distribution in the general population adjusted for sex, age and race [3,4,5]. The severity of the reduction depends on the gene involved, the mutation and the mode of inheritance of this disease [4]. The frequency of heterozygous FHBL in the general population is estimated to be 1:1000–1:3000 [4,6]. FHBL is caused mainly by mutations in the *APOB* gene which encodes the apolipoprotein B [2,7]. Less frequently, it is due to loss-of-function mutations in the *PCSK9* gene [2,6], a protagonist in cholesterol metabolism discovered in 2003 by identifying gain-of-function mutations in French families with familial hypercholesterolemia [8]. The *APOB* gene is located on the short arm of chromosome 2 at position 2p24.1 [9,10]. This gene encodes the apolipoprotein B that exists in the plasma in two isoforms: ApoB-100 which is the full-length protein secreted by the liver and associated with the very-low-density lipoprotein (VLDL) and its metabolic products: intermediate-density lipoprotein (IDL) and LDL, and ApoB-48 which is the intestinal variant associated with chylomicrons. ApoB-48 results from a tissue specific messenger RNA (mRNA) processing or editing in the intestine that converts codon 2153 to a stop codon [11,12,13]. More than 120 mutations in this gene have been reported to cause FHBL [7]. These mutations occur throughout the entire coding-region, mainly in exon 26, and are principally nucleotide substitutions (causing nonsense mutations) and deletions/insertions of a single or more nucleotides (causing frameshift mutations), and less commonly splice site mutations [3,4,14,15]. These mutations interfere with the full translation of the protein by introducing a premature stop codon in ApoB mRNA and causing the synthesis of short truncated proteins of different sizes, ranging from ApoB-2 to ApoB-89, as per a centile nomenclature (percent length of the native ApoB-100 molecule) [3,4,12]. Missense variants have also been identified [4,16]. The first missense mutation is “p.(Arg490Trp) previously reported as p.(Arg463Trp)” (c.1468C>T, rs771541567) by Burnett et al. in 2003 in an extended Lebanese family with FHBL living in Australia [16,17].

Patients who are heterozygous for mutations in the *APOB* gene are generally asymptomatic but present an increased risk of developing liver disease [18]. Truncations in the ApoB cause a defective synthesis and export of VLDL from the liver, which causes an accumulation of triglycerides (TG) in the hepatocytes [1], and thus exposes those patients to an increased risk of developing fatty liver disease (up to a 60% prevalence) [19]. Patients with homozygous or compound heterozygous FHBL present symptoms that are identical to those of abetalipoproteinemia caused by mutations in the microsomal triglyceride transfer protein (*MTP*) gene [4,11]. These principally include a fat malabsorption during the neonatal period, steatorrhea, vomiting, abdominal distension, failure to thrive, steatosis and deficiencies in fat-soluble vitamins. These symptoms improve after dietary fat restriction and supplementation with high doses of vitamins E and A. The progression of the disease can lead to atypical retinitis pigmentosa and spinocerebellar ataxia [4,11,12]. 

To our knowledge, no genetic study on FHBL has been conducted in the Middle East (ME) region. In fact, the ME region is characterized by a high prevalence of consanguineous marriages, metabolic syndrome, dyslipidemia characterized by low high-density lipoprotein cholesterol (HDL-C) levels and high triglycerides levels, familial hypercholesterolemia (FH), diabetes [20], and nonalcoholic fatty liver disease (NAFLD) [21]. More specifically, the Lebanese population is characterized by a high frequency of dyslipidemia, particularly FH [22]. Our team has conducted several studies on dyslipidemia in the Lebanese population that allowed to characterize the mutational spectrum of FH [22], to identify the first Lebanese mutation in the *LPL* gene causing type I hyperlipoproteinemia [23], and more recently to describe the first Lebanese pathogenic variant in the *ABCA1* gene causing Tangier disease [24]. In this article, patients were recruited as part of a molecular study aiming to investigate the genetic causes of FHBL in Lebanon to improve diagnosis and prevent associated risks especially of liver diseases. We characterize FHBL in three unrelated Lebanese families and four unrelated probands and highlight the presence of a frequent pathogenic variant in the *APOB* gene responsible for this phenotype, presumably as a result of a founder effect.

## 2. Results

### 2.1. Clinical Characteristics and Biochemical Analysis of the Probands and Their Relatives

**Figure 1:**
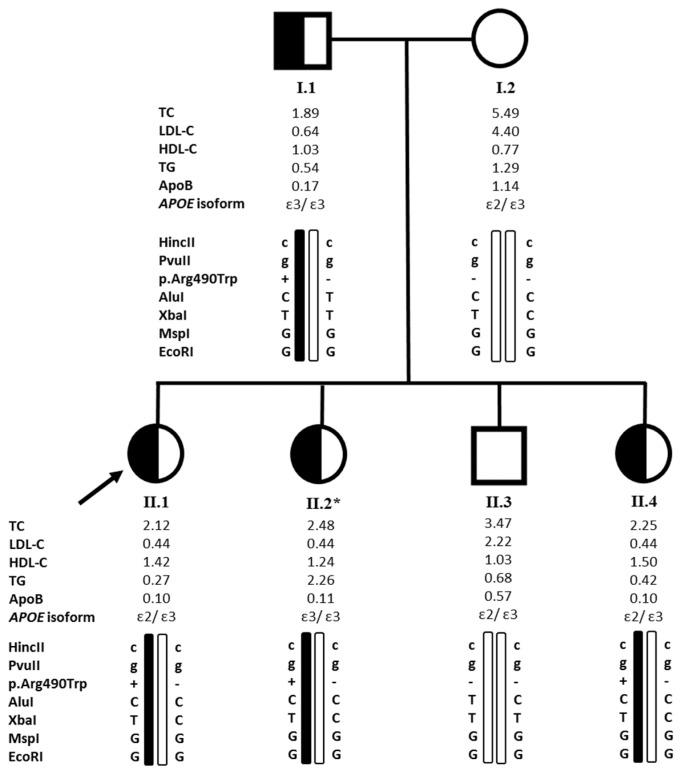
Pedigree of family 1 and haplotype analysis. The arrow indicates the proband. Half blackened symbols indicate heterozygous carriers of the p.(Arg490Trp) (p.(Arg463Trp), according to the original nomenclature) variant in the *APOB* gene and unblackened symbols indicate unaffected members. Filled bars indicate the allele carrying the mutation. The +/− sign indicates that the individual is heterozygous for the p.(Arg490Trp) variant and the −/− sign indicates that the individual does not carry the variant. *APOE* isoforms are also presented. TC, LDL-C, HDL-C, TG levels are given in mmol/L and ApoB levels are given in g/L. HDL-C: high-density lipoprotein cholesterol, LDL-C: low-density lipoprotein cholesterol, TC: total cholesterol, TG: triglycerides. The * sign indicates that the individual was not fasting when the blood sample was collected.

In the first family (Figure 1), the proband (II.1) was a 28-year-old Lebanese woman, referred to our laboratory by her endocrinologist at Hôtel Dieu de France hospital after perceiving very low levels of LDL-C and ApoB (0.44 mmol/L and 0.10 g/L, respectively). She was diagnosed at 32-years of age with fatty liver disease. Her father (I.1) aged 61 years also presented low levels of lipid parameters (LDL-C levels of 0.64 mmol/L and ApoB levels of 0.17 g/L) and was also diagnosed with fatty liver detected by ultrasonography. She had two sisters (II.2 and II.4) aged 25 and 16 years who also had low levels of lipid parameters (LDL-C levels of 0.44 mmol/L for both sisters and ApoB levels of 0.11 and 0.10 g/L, respectively) while her mother (I.2) (54 years old) and her brother (II.3) (21 years old) did not present low LDL-C nor ApoB levels (4.40 and 2.22 mmol/L for LDL-C levels and 1.14 and 0.57 g/L for ApoB levels, respectively) (Table 1). The father was recently diagnosed with Parkinson’s disease. Family history revealed that her father’s uncle had died from cirrhosis.

In the second family (Table 1), the proband (II.3) was a 47-year-old woman presenting low levels of LDL-C (1.50 mmol/L). Her mother (I.2) and sister (II.2) aged 73 and 52 years respectively also had low LDL-C levels (0.70 and 0.68 mmol/L, respectively) and suffered from diabetes and hypertension. The sister was treated with metformin, bisoprolol and valsartan. The mother had a breast cancer and was treated with tamoxifen. Her father (I.1) aged 93 years and brother (II.1) aged 53 years did not present hypocholesterolemia (LDL-C levels of 2.21 and 2.11 mmol/L, respectively) and suffered from diabetes (Table 1). The father also presented hypertension, prostate cancer, and dyslipidemia treated by simvastatin.

In the third family (Table 1), the proband (II.1) was a 60-year-old woman who presented low LDL-C levels (1.08 mmol/L). She suffered from diabetes with an HbA1c of 8.80% and a fasting blood glucose level of 11.93 mmol/L (2.15 g/L). Her father (I.1) aged 90 years was also diabetic and presented low LDL-C levels. He was diagnosed with multiple system atrophy disease six years ago.

In addition to these families, four unrelated probands were also recruited. Proband 4 was a 49-year-old male who presented low LDL-C levels (0.45 mmol/L) (Table 1) and was a smoker. He suffered from hepatic steatosis, diabetes and hypertension and was treated by metformin, losartan, hydrochlorothiazide, amlodipine and bisoprolol. Proband 5 was a 63-year-old woman who presented hypobetalipoproteinemia (LDL-C levels of 0.78 mmol/L) (Table 1) associated with a fatty liver revealed by an abdominal ultrasonography. She also suffered from diabetes, hypertension, osteoporosis, and complained about neuropathies. Her treatment consisted of glimepiride, glargine, metformin, dapagliflozine and irbesartan. An intestinal biopsy stained with Sudan black ruled out the presence of abetalipoproteinemia. Proband 6 was a 33-year-old woman with low LDL-C levels (1.38 mmol/L) (Table 1). Proband 7 was a 23-year-old woman with low levels of LDL-C (1.44 mmol/L) (Table 1).

### 2.2. Genetic Analysis

We sequenced exon 11 of the *APOB* gene, in which the pathogenic c.1468 C>T variation (rs771541567) has been previously reported by Burnett et al. in an extended Lebanese family living in Australia [16,17]. It was originally designated by p.(Arg463Trp) according to the original nomenclature [16] considering the amino acid numbering of the mature protein (without the signal peptide of 27 amino acids). Thus, it corresponds to p.(Arg490Trp) according to the present international nomenclature (the first amino acid of the ApoB being the initiator methionine NP_000375.3).

In the first family, sequencing of exon 11 revealed the presence of the p.(Arg490Trp) variant at the heterozygous state in the proband (II.1) and the affected relatives (I.1, II.2 and II.4) and its absence in non-affected individuals (I.2 and II.3), highlighting a segregation with the FHBL phenotype in the family (Figure 1). In the second family, the same variant was identified at the heterozygous state in the proband (II.3) and her sister (II.2) for whom the genetic testing was available (Table 1). In the third family, genetic testing was available for the proband (II.1) and her father (I.1) who were both heterozygous for the p.(Arg490Trp) variant (Table 1). Proband 4 and 5 were also heterozygous carriers (Table 1). The variant was not found in probands 6 and 7 and was absent in the 60 non-hypocholesterolemic individuals. In total, five out of the seven recruited probands, were heterozygous for the p.(Arg490Trp) variant, which represents approximately 70% of the cases of hypobetalipoproteinemia in the present FHBL cohort and was not found in the 60 non-hypocholesterolemic controls.

Interestingly, the amino acid arginine at position 490 is well conserved among species. The p.(Arg490Trp) variant was predicted to be disease causing on Mutation Taster (Grantham Matrix score of 101), deleterious on PROVEAN (with a score of −5.204), with probably damaging consequences on the functionality of the protein according to Polyphen-2 (score of 0.996, sensitivity: 0.36; specificity:0.97), and presented a CADD score of 34 suggesting that this variant is predicted to be among the top 0.1% of the most deleterious substitutions in the human genome. The allele frequency of this variant is estimated to be 7.96 × 10^−6^ in the general population according to GnomAD.

We also sequenced all the exons of the *PCSK9* gene in which loss-of-function mutations have been associated with hypocholesterolemia [25]. The sequencing revealed that one proband (Proband 5) and the sister of Proband II.3 in family 2 (II.2) were heterozygous for the common polymorphism p.Leu21dup in exon 1 that lowers LDL-cholesterol levels [22] (Table 1). 

We also sequenced exon 4 of the *APOE* gene knowing that the ApoE isoforms may be responsible for a 15–60% of LDL-C plasma levels variation in FHBL subjects versus 10% in the general population [26,27]. *APOE* sequencing revealed that in family 1, some individuals presented the E2/E3 isoforms (I.2, II.1, II.3, and II.4) while others presented the E3/E3 isoforms (I.1 and II.2) (Figure 1). The sequenced members of family 2 (II.2 and II.3) and family 3 (I.1 and II.1), as well as the four other probands (probands 4, 5, 6 and 7) presented the E3/E3 isoforms (Table 1).

### 2.3. The Founder p.(Arg490Trp) Variant

**Figure 2:**
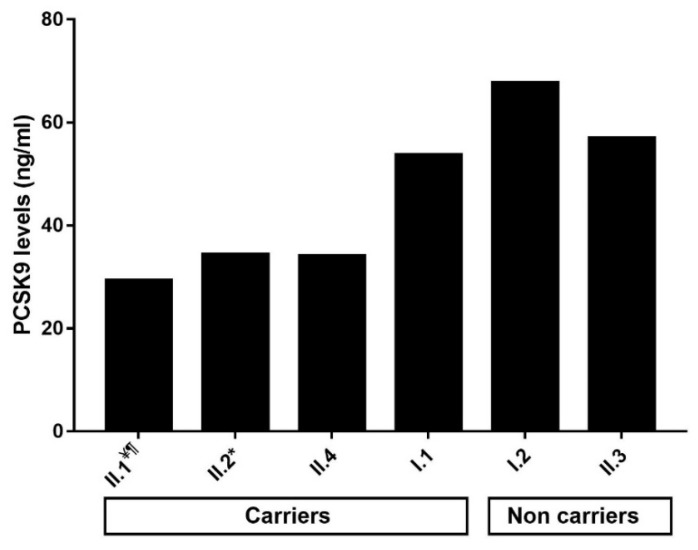
Levels of circulating PCSK9 in the carriers of the p.(Arg490Trp) variant and the non-carriers in family 1. ^¥^ Indicates the proband in the family. ^¶^ Indicates that PCSK9 levels were measured in the serum because plasma was not available and that the value was dismissed while calculating the mean and the standard deviation in plasma. * Indicates that the subject was not fasting when the blood sample was collected.

The relatively high frequency of the p.(Arg490Trp) variant in the present FHBL cohort and its occurrence in Lebanese families living abroad raised questions about its origin and the presence of a founder effect. Therefore, we conducted a haplotype analysis to determine whether the chromosomal background of the variation detected in some individuals is identical. The latter showed that in families 1 (Figure 1), 2 and 3, all the individuals harboring the p.(Arg490Trp) variant, as well as probands 4 and 5, carry a similar mutant haplotype (Table 2).

For the HincII and PvuII markers, all heterozygous carriers presented at both sites a nucleotide substitution which is predominant compared to the wild type nucleotide, with a prevalence of 86% and 93% respectively. For the AluI marker, all heterozygous individuals did not present a polymorphism at this site, the wild type amino acid Ala was conserved, with a prevalence of 47%. For the XbaI marker, all heterozygous individuals had a nucleotide substitution, with a prevalence of almost 50%. For the MspI and EcoR1 markers, all heterozygotes had the wild type amino acid conserved. The prevalence of the wild type amino acid is respectively around 90% and 81%. These prevalences are reported in Non-Finnish European according to GnomAD and in an Austrian population reported in previous studies [28].

### 2.4. Impact of the p.(Arg490Trp) Mutation on Plasma PCSK9 Levels

We measured plasma and serum PCSK9 levels in family 1 and in some of the other individuals of the present cohort, for whom plasma was available. The results are presented in Table 1 and Figure 2. In family 1, heterozygous carriers of the p.(Arg490Trp) variant (I.1, II.1, II.2, and II.4) presented an approximately 35% decrease in plasma levels of PCSK9 compared to non-affected individuals (I.2 and II.3) (mean ± standard deviation of 40.71 ± 11.25 ng/mL versus 62.38 ± 7.58 ng/mL, respectively). In addition, we found a significant positive correlation between circulating PCSK9 and ApoB levels in all studied probands and their family members (r = 0.81; *p* = 0.004) as well as in FHBL patients carrying the p.(Arg490Trp) variant (r = 0.85; *p* = 0.02).

## 3. Discussion

This is the first study conducted to investigate the genetic causes of hypobetalipoproteinemia in Lebanon, and to measure PCSK9 levels in familial hypobetalipoproteinemia caused by a missense mutation. We searched for the p.(Arg490Trp) variant which was described for the first time in 2003 by Burnett et al. in an extended family with FHBL of Lebanese origin living in Australia [16,17]. This variant has a very rare frequency in GnomAD and is predicted to be pathogenic according to in silico genetic tools. Moreover, it had been detected in several unrelated hypocholesterolemic families in Italy and Canada and in individuals from Spanish and Dutch origin [3,26,31,32,33,34]. Interestingly, the family from Australia [16] and of the one from Canada [32], originated from Lebanon. In the present cohort, we found that the p.(Arg490Trp) was present in 71% of the hypocholesterolemic probands, segregated with the FHBL disease in the studied families, and was not found in 60 Lebanese controls. Thus, this variant seems a cause of FHBL in the Lebanese patients. Haplotype analysis was performed and showed that the FHBL patients harboring the p.(Arg490Trp) pathogenic variant shared as well surrounding genetic variants in a common haplotype, despite having different geographical origins within Lebanon. A founder mutation transmitted by a common ancestor is thus suspected but needs larger studies to be confirmed. Nevertheless, our results highlight the importance of investigating the genetic causes of FHBL in Lebanon, starting with the search for the p.(Arg490Trp) variant in exon 11 of *APOB*.

Moreover, two carriers of the p.(Arg490Trp) were heterozygous for the E2 allele in the *APOE* gene and two others for the p.Leu21dup in exon 1 of the *PCSK9* gene. The presence of a single E2 allele is thought to be associated with a ≈ 10 mg/dL reduction in TC compared to people with other *APOE* allele combinations [35], and the presence of the p.Leu21dup polymorphism is associated with lower cholesterol levels in populations with normal to low LDL-cholesterol levels, but also in hypercholesterolemic patients [22]. While no other mutation in the *PCSK9* gene was detected, we observed that there was a significant decrease in PCSK9 levels in affected individuals of family 1 compared to the unaffected. These results are consistent with the work of Fazio et al., who studied the association between plasma PCSK9 levels and low LDL-C levels in subjects with FHBL due to truncations in the ApoB protein and found that PCSK9 levels were decreased in subjects harboring a truncating mutation [36].

The pathogenicity of this variant has been proven in both in vitro and in vivo functional studies [16,26]. It might be a result of an enhanced binding to MTP and a retention of the protein in the endoplasmic reticulum, causing a defective lipidation, assembly and secretion of ApoB-containing lipoproteins, resulting in the development of hypobetalipoproteinemia [3,4,16,26]. Mean values of TC, LDL-C, HDL-C, and TG in FHBL heterozygous patients carrying the p.(Arg490Trp) variant included in this study, as well as the presence of fatty liver, were comparable to those reported in the literature in patients harboring the same variant [3,16,17,26,31,32]. In fact, it is known that subjects with FHBL caused by truncating variants in the *APOB* gene are at increased risk to develop fatty liver because of a defective synthesis and export of VLDL from the liver, which causes an accumulation of TG in the hepatocytes [1]. In the present study, four carriers of the p.(Arg490Trp) variant suffered from fatty liver disease and the uncle of one carrier had died from cirrhosis. Other cases of liver disease in carriers of the p.(Arg490Trp) variant were reported in the literature with either a significant increase in liver enzymes when liver ultrasonography was not performed or hepatic steatosis [3,16,26,32,33,34], with mild to severe forms reported in children aged 9 and 15 years [26]. Moreover, a case of NASH and liver cirrhosis with esophageal varices had been reported in a FHBL patient and his sister respectively, both harboring this variant. Interestingly, the sister who also presented hypobetalipoproteinemia suffered from type 2 diabetes [32]. Relationships between these two latest conditions have been largely investigated. A review article which was looking for a relationship between diabetes and FHBL found that the latter condition resulting from *APOB* gene variants does not seem to cause an increase in the risk of diabetes mellitus (DM) [37]. Moreover, many studies aimed to assess the relationship between hepatic steatosis caused by FHBL and insulin resistance and concluded that these two factors are not linked, even in animal models [38,39,40]. In our study, five individuals carrying the p.(Arg490Trp) variant were diabetic. It is uncertain whether the high frequency of diabetes among the present FHBL cohort harboring the p.(Arg490Trp) variant is due to the variant itself or to the high incidence of diabetes in the Lebanese population [41]. Therefore, in patients with FHBL, liver enzymes should be regularly monitored, and liver ultrasonography should be performed if these were elevated [42].

Another interesting observation is that three individuals from the present cohort carrying the p.(Arg490Trp) suffered from a neurological manifestation ranging from neuropathies to Parkinson’s disease and multiple system atrophy. It is not clear whether the neurological disorders developed by these individuals are caused by FHBL or by other factors. In fact, Parkinson’s disease [43] and a case of a severe late onset neurological disorder had been described in patients with hypobetalipoproteinemia [44], although neurological disorders and central nervous system deterioration occur mainly with abetalipoproteinemia and homozygous or compound heterozygous FHBL [3,12]. Thus, it may be essential to perform a neurological clinical evaluation on a regular basis, even in heterozygous FHBL patients, in order to instore the early appropriate treatment, and in some cases to recommend a moderate-dose vitamin E supplementation in individuals with low serum vitamin E concentration. The early diagnosis of these forms of serious late onset complications is necessary because these latter are usually impossible to stop, once they have been developed by the patient [11,12,44].

## 4. Materials and Methods

### 4.1. Study Participants

Three unrelated families and four unrelated probands from different regions in Lebanon were recruited in collaboration with endocrinologists and gastroenterologists at the Endocrinology and Gastroenterology Clinics of Hôtel Dieu de France hospital in Beirut upon perceiving permanent low LDL-C levels (Table 1). Inclusion criteria were LDL-C levels below 1.55 mmol/L, without any secondary causes such as lipid-lowering medications, malnutrition, hepatic problems, cancer, hyperthyroidism or others [45]. For each patient, we collected the clinical and familial history. On the other hand, 60 Lebanese adult volunteers were recruited as a control group to assess the presence of the variation in the general population. Informed consent was obtained from all subjects involved in the study. The study was conducted according to the guidelines of the Declaration of Helsinki, and approved by the Ethics Committee of Hôtel Dieu de France Hospital and Saint Joseph University of Beirut (CEHDF140).

### 4.2. Laboratory and Biochemical Tests

Blood samples were obtained after the subjects had fasted overnight, plasma and serum were prepared. Lipid measurements were determined on a COBAS INTEGRA^®^ analyzer (Roche Diagnostics, Basel, Switzerland).

### 4.3. DNA Analysis and Variant Detection

Genomic DNAs of all the participants were extracted from peripheral blood leukocytes using Flexigene^®^ DNA Kit from Qiagen (Hilden, Germany) according to the manufacturer’s instructions. The exon 11 of the *APOB* and all exons of the *PCSK9* genes and their flanking exon-intron boundaries, as well as the region containing the two SNPs for genotyping the *APOE* gene [46] were amplified by polymerase chain reaction (PCR) and sequenced using the Sanger method. All the primers’ sequences and PCR conditions are available upon request. For DNA sequence assembly and variant detection, we used the CodonCode Aligner^®^ Software.

### 4.4. In Silico Analysis of the Variant

For frequency determination of the variant, we used the Genome Aggregation database (gnomAD; http://gnomad.broadinstitute.org/, accessed on 7 April 2021), and for pathogenicity prediction we used Polymorphism Phenotyping version 2 (PolyPhen-2; http://genetics.bwh.harvard.edu/pph2/, accessed on 7 April 2021), Protein Variation Effect Analyzer (Provean; http://provean.jcvi.org/index.php, accessed on 7 April 2021), Mutation Taster (http://www.mutationtaster.org/, accessed on 7 April 2021), and the Combined Annotation Dependent Depletion score (CADD score; https://cadd.gs.washington.edu/snv, accessed on 7 April 2021).

### 4.5. Haplotype Analysis

We conducted a haplotype analysis using a set of diallelic markers described in the literature [28]. Six common polymorphisms within the *APOB* gene caused by single base substitutions in various exons and introns distributed all over the gene [28] were amplified by PCR and sequenced using the Sanger method. All the primers’ sequences and PCR conditions are available upon request. 

These diallelic markers resulted from the following substitutions in *APOB* gene (according to the present nomenclature of the apolipoprotein B) that modify a recognition site by a specific restriction enzyme: p.Ala618Val (AluI), p.Thr2515 (XbaI), p.Arg3638Gln (MspI), p.Glu4181Lys (EcoRI), and two occurring in intron 4 (HincII and PvuII) [28]. 

### 4.6. PCSK9 Measurements

We measured plasma and serum PCSK9 levels using a commercial ELISA kit (Human Proprotein Convertase 9/PCSK9 Duoset catalogue no. DY3888; R&D Systems, Minneapolis, MN, USA) and the Bio-Plex Pro assay technology (Luminex Corporation, Austin, TX, USA) as previously described [47].

## 5. Conclusions

In this article, we highlight the presence of a pathogenic variant, p.(Arg490Trp), in exon 11 of the *APOB* gene, in several Lebanese families and probands presenting with FHBL (in approximately 70% of the probands), presumably because of a founder mutation. Therefore, in the case of a patient presenting with primary hypobetalipoproteinemia or with a hepatic steatosis without any risk factors and low LDL-C levels, family investigation should be conducted in order to identify a familial aspect of the hypobetalipoproteinemia. Molecular diagnosis should be implemented by searching firstly for the p.(Arg490Trp) variant in exon 11 of the *APOB* gene. Moreover, these patients and their families should be advised on the risk of consanguineous marriage, especially that its prevalence in Lebanon was of 35.5% [48], knowing that homozygous FHBL can lead, among other complications, to serious central nervous system injuries that can lead to death if not treated early [12]. In conclusion, genetic investigation of FHBL as well as dyslipidemia in Lebanon and the Middle East is crucial in order to improve patient care, ensure an early management of the disease, and prevent its complications.

## Figures and Tables

**Table 1:** Lipid measurements, clinical history (diabetes, liver disease and neurological problems) and genetic characteristics in the study population.

Subject	Age	Gender	TC	HDL-C	TG	LDL-C ^§^	LDL-C ^#^	ApoB (g/L)	Plasma PCSK9 Levels (ng/mL)	Fatty Liver	Diabetes	Neurological Problem	HBL	*APOE* Isoforms	p.Leu21dup in *PCSK9*	p.(Arg490Trp) in *APOB*
			**(mmol/L)**													
Family 1																
I.1	61	M	1.89	1.03	0.54	0.64		0.17	53.70	Yes	No	Parkinson’s disease	Yes	ε3/ε3	No	Yes
I.2	54	F	5.49	0.77	1.29	4.40		1.14	67.74	No	No		No	ε2/ε3	No	No
II.1 ^¥^	28	F	2.12	1.42	0.27	0.44		0.10	29.36 ^¶^	Yes	No		Yes	ε2/ε3	No	Yes
II.2 *	25	F	2.48	1.24	2.26	0.44		0.11	34.41	No	No		Yes	ε3/ε3	No	Yes
II.3	21	M	3.47	1.03	0.68	2.22		0.57	57.02	No	No		No	ε2/ε3	No	No
II.4	16	F	2.25	1.50	0.42	0.44		0.10	34.01	No	No		Yes	ε2/ε3	No	Yes
Family 2																
I.1	93	M	4.19	0.98	2.21		2.21	N/A		N/A	Yes		No	N/A	N/A	N/A
I.2	73	F	1.84	0.93	0.47		0.70	N/A		N/A	Yes		Yes	N/A	N/A	N/A
II.1	53	M	4.16	1.73	0.71		2.11	N/A		N/A	Yes		No	N/A	N/A	N/A
II.2	52	F	2.59	1.14	1.69		0.68	0.45	68.35	No	Yes		Yes	ε3/ε3	Yes	Yes
II.3 ^¥^	47	F	2.72	0.88	0.75		1.50	0.40	34.16	No	No		Yes	ε3/ε3	No	Yes
Family 3																
I.1	90	M								No	Yes	Multiple System Atrophy	Yes	ε3/ε3	No	Yes
II.1 ^¥^	60	F	2.69	1.08	0.93	1.08		0.41	68.98	No	Yes		Yes	ε3/ε3	No	Yes
Proband 4	49	M	1.60	0.88	0.59		0.45	N/A		Yes	Yes		Yes	ε3/ε3	No	Yes
Proband 5	63	F	2.38	0.96	1.41		0.78	N/A		Yes	Yes	Neuropathies	Yes	ε3/ε3	Yes	Yes
Proband 6	33	F	3.96	2.43	0.34		1.38	N/A		N/A	No		Yes	ε3/ε3	No	No
Proband 7	23	F	3.21	1.47	0.67		1.44	0.66	69.39	N/A	No		Yes	ε3/ε3	No	No
**Mean ± SD p.(Arg490Trp)** **carriers** **(*n* = 9)**			2.30 ± 0.38	1.13 ± 0.22	0.83 ± 0.50	0.72 ± 0.36		0.25 ± 0.16	48.94 ± 17.05							

F: female, M: male, HBL: hypobetalipoproteinemia; HDL-C: high-density lipoprotein cholesterol, LDL-C: low-density lipoprotein cholesterol, TC: total cholesterol, TG: triglycerides, N/A: not available, SD: standard deviation. ^§^ Indicates that LDL-C was measured by a direct method. ^#^ Indicates that LDL-C value was calculated using the Friedewald formula. ^¥^ Indicates the proband in the family. ^¶^ Indicates that PCSK9 levels were measured in the serum because plasma was not available and that the value was dismissed while calculating the mean and the standard deviation in plasma. * Indicates that the individual was not fasting when the blood sample was collected and that the TG value was dismissed while calculating the mean and SD of TG levels.

**Table 2:** Mutant haplotypes of the affected probands and relatives harboring the p.(Arg490Trp) (p.(Arg463Trp) according to the original nomenclature) variant.

					Haplotype of the APOB-p.(Arg490Trp) Carriers				
Haplotype Marker	Reference SNP [29,30]	Nucleotide Change (NM_000384.3)	Amino Acid Change (Original/Present Nomenclature NP_000375.3)	Prevalence in Non-Finnish European (GnomAD)	Proband (II.1) and Affected Members of Family 1	Proband (II.3) and Member (II.2) of Family 2	Proband (II.1) and Member (I.1) of Family 3	Proband 4	Proband 5
HincII			Intron 4		c	c	c	c	c
PvuII			Intron 4		g	g	g	g	g
Heterozygous for p.(Arg490Trp)	rs771541567	c.1468C>T	p.(Arg463Trp)/p.(Arg490Trp)	0.000017	T	T	T	T	T
AluI	rs679899	c.1853C>T	p.(Ala591Val)/p.(Ala618Val)	0.47	C	C	C	C	C
XbaI	rs693	c.7545C>T	p.(Thr2488=)/p.(Thr2515=)	0.499	T	T	T	T	T
MspI	rs1801701	c.10913G>A	p.(Arg3611Gln)/p.(Arg3638Gln)	0.091	G	G	G	G	G
EcoRI	rs1042031	c.12541G>A	p.(Glu4154Lys)/p.(Glu4181Lys)	0.182	G	G	G	G	G

## Data Availability

All the data have been included in article.
